# Combination Therapy with Doxorubicin-Loaded Reduced Albumin Nanoparticles and Focused Ultrasound in Mouse Breast Cancer Xenografts

**DOI:** 10.3390/ph13090235

**Published:** 2020-09-07

**Authors:** Daehyun Kim, Seung Soo Lee, Woo Young Yoo, Hyungwon Moon, Aesin Cho, So Yeon Park, Yoon-Seok Kim, Hyun Ryoung Kim, Hak Jong Lee

**Affiliations:** 1 Department of Nano Science and Technology, Graduate School of Convergence Science and Technology, Seoul National University, Seoul 151-744, Korea; daehyun.kim@nanoimgt.com; 2 Department of Radiology, Seoul National University Bundang Hospital, 82 Gumi-ro 173, Bundang-gu, Seongnam 13620, Korea; 3 IMGT Co., Ltd., Seongnam 13605, Korea; 2slee88@gmail.com (S.S.L.); wooyoung.yoo@nanoimgt.com (W.Y.Y.); hyungwon.moon@nanoimgt.com (H.M.); aeshin.cho@nanoimgt.com (A.C.); soyeon.park@nanoimgt.com (S.Y.P.); yoonseok.kim@nanoimgt.com (Y.-S.K.); hyunryoung.kim@nanoimgt.com (H.R.K.)

**Keywords:** breast cancer, doxorubicin, albumin nanoparticles, focused ultrasound, drug delivery

## Abstract

Because chemotherapeutic drugs are often associated with serious side effects, the central topic in modern drug delivery is maximizing the localization of drugs at the target while minimizing non-specific drug interactions at unwanted regions. To address this issue, biocompatible nanoparticles have been developed to enhance the drug half-life while minimizing the associated toxicity. Nevertheless, relying solely on the enhanced half-life and enhanced permeability and retention (EPR) effects has been ineffective, and designing stimulus-sensitive nanoparticles to introduce the precise control of drug release has been desired. In this paper, we introduce a pH-sensitive, reduced albumin nanoparticle in combination with focused ultrasound treatment. Not only did these nanoparticles have superior therapeutic efficacy and toxicity profiles when compared to the free drugs in xenograft mouse models, but we were also able to show that the albumin nanoparticles reported in this paper were more suitable than other types of non-reduced albumin nanoparticles as vehicles for drug delivery. As such, we believe that the albumin nanoparticles presented in this paper with desirable characteristics including the induction of strong anti-tumor response, precise control, and superior safety profiles hold strong potential for preclinical and clinical anticancer therapy.

## 1. Introduction

Breast cancer remains one of the most common cancers in women. It is the second most common cancer among American women, with 13% of the entire female population facing the risk of cancer development some time in their lives [1]. The receptor status of breast cancers is used in clinics to identify the subtypes and subsequent treatment plans [2]. For example, epidermal growth factor 2 (ERBB2/HER2) is a transmembrane receptor tyrosine kinase that is overexpressed in approximately 20% of all breast cancer patients. ERBB2-targeted therapies such as anti-ERBB2 antibodies (such as trastuzumab or pertuzumab) or tyrosine kinase inhibitors (such as lapatinib and neratinib) have been successfully used against these types of breast cancers [2,3]. Nevertheless, for patients that are diagnosed with triple-negative subtypes—those that do not express genes for estrogen, progesterone, and ERBB2 receptors—the anticipated outcome and overall survival (OS) remains much lower (OS of 10–13 months vs. 5 years for *ERBB2*+) due to the lack of tumor-specific markers and their aggressive nature [2,4]. The standard of care for triple-negative breast cancer is neoadjuvant chemotherapy, in which drugs such as doxorubicin (DOX) are administered prior to the surgical removal of the tumor [2,5,6]. However, these cytotoxic drugs fail to discriminate between normal and cancerous cells, preventing the use of a large dose due to potential systemic toxicity.

Nanoparticle formulations such as liposomal doxorubicin (Doxil) and albumin-bound paclitaxel (Abraxane) have been developed to overcome such issues. In particular, Abraxane has gained great interest because it was the first commercialized nanoformulation that utilized fully biocompatible, nontoxic, and nonimmunogenic serum proteins for enhancing the therapeutic index. As such, since the advent of albumin nanoparticles, different groups have investigated various methods of albumin nanoparticle formulation [7]. Of such methods, the desolvation method had gained wide popularity due to the simplicity in production with predictable outcomes [8,9]. During the desolvation process, albumins are crosslinked with agents such as glutaraldehyde (GTA) to yield uniform, spherical nanoparticles with negative surface charges. In addition to using GTA as a crosslinker, carbodiimides [10] and thiolated albumins that form additional disulfide crosslinking [11] have been also evaluated. Nevertheless, the presence of open-armed crosslinkers such as the free aldehyde group present on GTA may elicit potential toxicity or non-specific interactions, preventing the full release of the therapeutic payload. Therefore, we have developed a modified albumin nanoparticle with a reduced surface, which still maintains the important qualities associated with albumin nanoparticles as a vehicle while minimizing active functional groups to eliminate the potential complications described above.

Based on a meta-analysis, less than 0.7% of the administered nanoparticle dose is found to be delivered to the tumor of interest [12]. Therefore, not only is developing a biocompatible drug delivery platform that can selectively release the cargo at the target of interest important, but implementing a method to improve the drug and nanoparticle accumulation at the target to further complement the enhanced permeability and retention (EPR) effect is just as important. To address this issue, the use of focused ultrasound as a method to enhance drug delivery has gained great interest in recent years. This technique has been used to temporarily disrupt the blood–brain barrier (BBB), creating vascular fenestrations for small molecules to extravasate and enter the central nervous system (CNS) [13,14]. In addition, this concept of creating temporary openings has been applied to internal tumors with poor vascularization and high interstitial pressure, allowing the better accumulation of drugs and nanoparticles of interest at the target [15,16,17].

In this study, we developed acid-sensitive DOX-loaded albumin nanoparticle formulations combined with focused ultrasound treatment for preferential accumulation at the target of interest and competent drug release. Unlike conventional desolvation-based albumin nanoparticles, our reduced albumin nanoparticles fully release the therapeutic cargo at acidic pH as found in the tumor microenvironment and intracellular compartments such as the lysosome. By enhancing the EPR effect, we were also able to increase the accumulation of nanoparticles at the tumor site, which resulted in an improved therapeutic index.

## 2. Results

### 2.1. Schematics of Albumin Nanoparticle Synthesis

**Figure 1:**
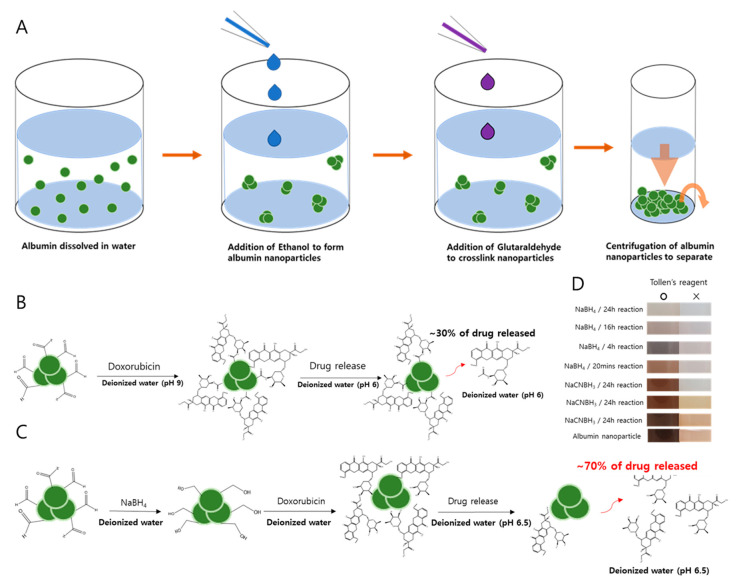
The synthesis and reduction protocols for albumin nanoparticles. (**A**) The desolvation method proposed by Langer et al. [18] is modified in the synthesis of albumin nanoparticles. (**B**) Speculated reaction of aldehyde–doxorubicin (DOX). (**C**) Schematic diagram of the interaction between reduced albumin nanoparticles (rAlb-NPs) and DOX after reducing aldehydes to alcohol. (**D**) Detection of aldehydes was performed by the Tollens’ test to confirm the removal of unreacted aldehyde from the surface of the nanoparticles.

A summary of albumin nanoparticle synthesis is described in Figure 1 based on the method proposed by Langer et al [18]. Human serum albumin (HSA) is first dissolved in water, then ethanol is added to form albumin nanoparticles. Once the nanoparticles form, crosslinkers such as GTA are used to crosslink nanoparticles into stable forms (Figure 1A). While DOX is added directly to the nanoparticles in the original method (Figure 1B), we had reduced the albumin nanoparticles first with sodium borohydride before the addition of DOX to ensure that the unreacted aldehydes are converted into unreactive alcohols (Figure 1C). Such a step was necessary to prevent covalent bonding between DOX and nanoparticles and to facilitate improved drug release.

### 2.2. Characterization of the Albumin Nanoparticles

**Figure 2:**
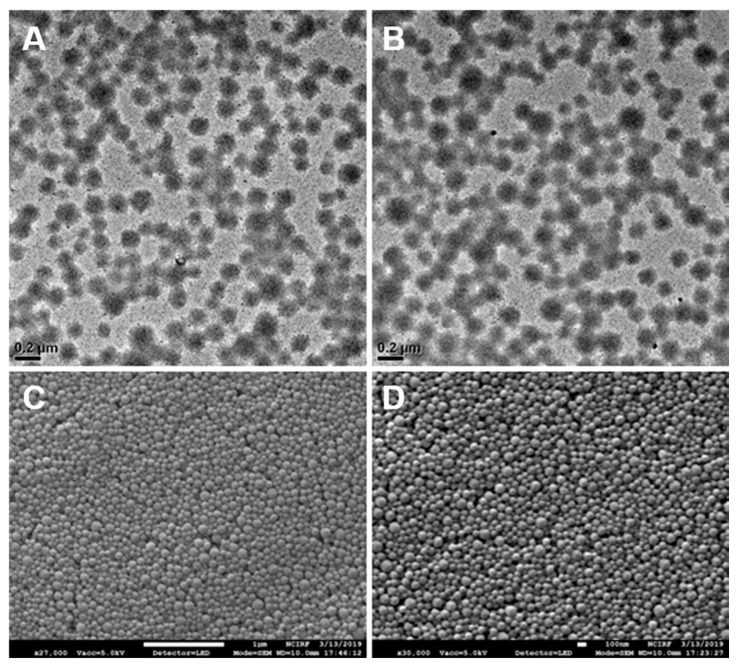
Images of Alb-NPs and rAlb-NPs obtained by electron microscopy. Based on the analysis of the images, the two nanoparticles had similar spherical morphology and size. (**A**) TEM images of rAlb-NPs. (**B**) TEM images of Alb-NPs. (**C**) SEM images of rAlb-NPs. (**D**) SEM images of Alb-NPs.

The albumin nanoparticles (Alb-NPs) based on the desolvation method and reduced albumin nanoparticles (rAlb-NPs) were characterized according to size and zeta potential (Table 1). For size and zeta potential measurements, the dynamic light scattering technique was used (Malvern Zetasizer Nano, Malvern Instrument Ltd., Malvern, UK). There were no significant differences between the sizes and the total yields of the nanoparticle formulations, with the mean diameter of the Alb-NPs being 146 ± 37.5 nm and that of the rAlb-NPs being 142 ± 31.1 nm. The sizes and the shapes of the nanoparticles were also evaluated with SEM and TEM images (Figure 2), which showed a group of spherical nanoparticles that were well-dispersed across the medium. However, it was observed that the zeta potential values of the Alb-NPs were more extreme than those of rAlb-NPs, with each having voltages of −51.3 ± 2.1 and −24.4 ± 2.8 mV, respectively. We speculated that the functional groups on the surface of the Alb-NPs contributed to the negative charges, and the subsequent reduction of the surface functional groups led to increased zeta potential of reduced albumin nanoparticles. In addition, the stability of the nanoparticles was monitored for up to 6 months. At designated times, aliquots of the nanoparticles were removed from 4 °C storage, and their size and zeta potential were measured using the DLS technique. The sizes and zeta potentials of the nanoparticles remained consistent, suggesting their excellent stability in solution (Table 2). Furthermore, the rAlb-NPs were checked for purity using inductively coupled plasma atomic transmission spectroscopy (ICP-AES), where chemical elements such as boron could be detected. According to the ICP data, 31.17 ppm of boron was detected per 60 mg/mL of rAlb-NPs, which would be considered as negligible and non-toxic to the human body [19].

The three types of DOX-loaded Alb-NPs were then characterized. According to the DLS measurements, the mean diameters of the DOX-loaded Alb-NPs were 151 ± 21.5 nm for cDOX, 144 ± 28.2 nm for sDOX, and 146 ± 31.8 nm for rDOX (Table 1). Based on the data, the presence of doxorubicin on the surface of the nanoparticles did not affect the overall size of the Alb-NPs. Nevertheless, the zeta potential of sDOX (from Alb-NPs) was smaller (−21.6 ± 1.6 mV) than that of rDOX (from rAlb-NPs, −14.7 ± 0.8 mV), which corresponds to the zeta potential measurements of the parent albumin nanoparticles.

### 2.3. Loading and Release Kinetics of cDOX, sDOX, and rDOX

Next, the loading and release kinetics of DOX-loaded albumin nanoparticles were evaluated. For cDOX, the “encapsulation” efficiency was calculated differently because DOX was added to the nanoparticles during the desolvation process. A DOX/human serum albumin (HSA) ratio of 1:10 (*wt*/*wt*) was used throughout the entire process because higher DOX concentrations (1:1, 1:3, and 1:5 DOX to HSA ratios) caused the zeta potential values to approach 0 mV, inducing aggregation of the nanoparticles. Accordingly, the loading efficiency of DOX on cDOX, sDOX, and rDOX was 67.1 ± 5.87%, 95.2 ± 5.21%, and 95.1 ± 3.51% at the 1:10 ratio, respectively, when the supernatants were analyzed using HPLC (Table 3).

**Figure 3:**
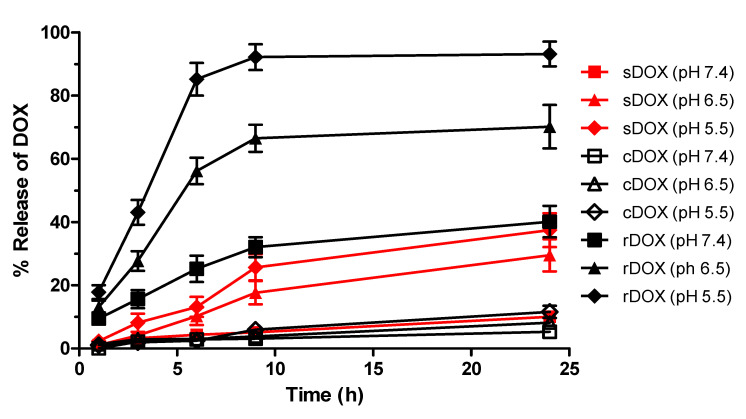
The pH-dependent release of DOX from albumin nanoparticles. rDOX with minimal non-specific interactions had improved release kinetics compared to cDOX or sDOX, affirming the results from DOX extraction. Values are mean ± SD (*n* = 3).

We also studied the release kinetics for the drug payloads from the albumin nanoparticles under acidic conditions and the stimulus of ultrasound. We speculated that with increased protons in the environment, the release of DOX would be favored as the pH became more acidic (e.g., in the tumor microenvironment) and the electrostatic interaction between the drug and the nanoparticle became reversed to the point where almost all the DOX would be released in extremely acidic environments. Therefore, the profiles of DOX release from the nanoparticles were evaluated at multiple pHs. Surprisingly, we observed that for cDOX and sDOX, a maximum of 40% of the loaded drug was released regardless of how acidic the environment was, while approximately 60% of the drug remained bound to the nanoparticles (Figure 3). Nonetheless, the release kinetics were much better for rDOX, with more than 93% of the drug being released after 24 h. We then tried to extract the DOX bound to the nanoparticles by decreasing the pH of the release media to 1. Nevertheless, only a slight increase in DOX was detected after extraction (Table 3). We speculated that the presence of free aldehyde arms from the GTA crosslinker and non-specific binding pockets of the albumin could induce non-specific binding with the amine group present on DOX molecules, inducing a strong, irreversible linkage. To further develop this hypothesis, we used Tollens’ reagent to evaluate the presence of active aldehydes on the albumin nanoparticles. According to the colorimetric analysis, we were able to observe that the unreduced Alb-NPs had a strong presence of reactive aldehydes on their surface, while using stronger reducing agents (sodium borohydride instead of sodium cyanoborohydride for a longer period) led to a near-complete elimination of the aldehyde groups on the nanoparticles without strongly affecting their properties (Figure 1D).

### 2.4. In Vitro Cell Viability Study and Confocal Microscopy

**Figure 4:**
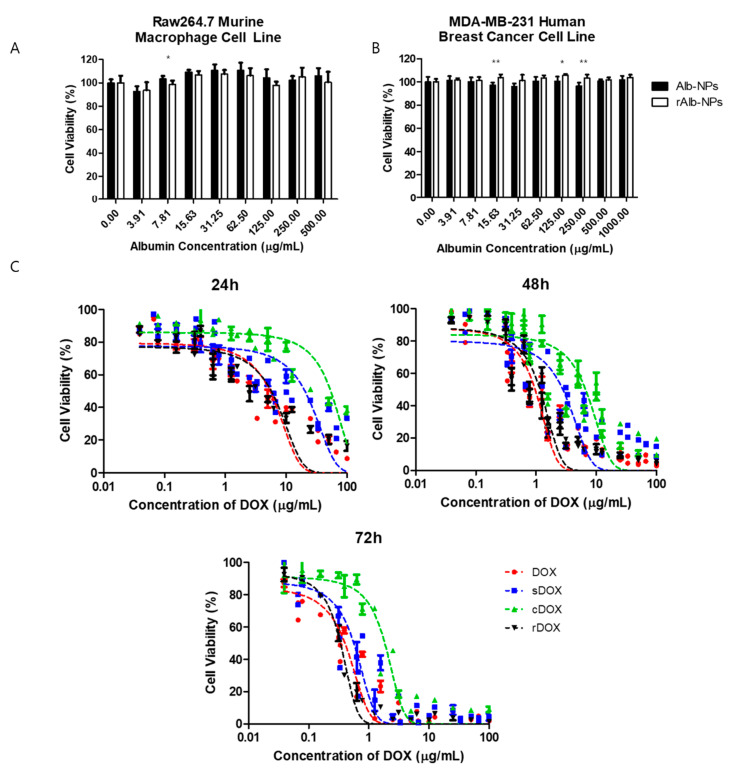
Cytotoxicity of albumin nanoparticles and drug-loaded albumin nanoparticles in vitro. First, the cytotoxicity of the vehicles (Alb-NPs and rAlb-NPs) were evaluated using (**A**) Raw264.7 murine macrophage cells and (**B**) MDA-MB-231 human breast cancer cells at different nanoparticle concentrations. (**C**) The cytotoxicity of DOX, sDOX, cDOX, and rDOX at various time points was compared, and the IC_50_ values were calculated. Values are mean ± SD (*n* = 6). * *p* ≤ 0.05, ** *p* ≤ 0.01.

The cytotoxic effects of albumin nanoparticles and DOX-loaded nanoparticles were first evaluated in vitro using the Raw264.7 murine macrophage cell line and MDA-MB-231 human breast cancer cells. First, the effects of bare albumin nanoparticles on the cell viability of Raw264.7 cells were examined by incubating different concentrations of Alb-NPs and rAlb-NPs with the cells. It was observed that both Alb-NPs and rAlb-NPs did not have a significant effect on Raw264.7 (Figure 4A) and MDA-MB-231 (Figure 4B) cell viability and morphology when up to 500 μg/mL (HSA concentration) of the nanoparticles were tested. Furthermore, the cytotoxic effects of DOX-loaded sDOX, cDOX, and rDOX were compared with those of the free drug, and the IC_50_ values were obtained. At 24 h post-incubation, the IC_50_ values of DOX, sDOX, cDOX, and rDOX were 5.24 ± 0.67, 20.36 ± 3.73, 54.04 ± 7.87, and 5.69 ± 0.85 μg/mL (DOX concentration), respectively, while at 48 h and 72 h post-incubation, the IC_50_ values were 0.96 ± 0.09, 2.79 ± 0.41, 6.66 ± 0.93, and 1.17 ± 0.11 μg/mL at 48 h and 0.40 ± 0.03, 0.56 ± 0.06, 1.84 ± 0.19, and 0.34 ± 0.02 μg/mL at 72 h for DOX, sDOX, cDOX, and rDOX, respectively (Figure 4C). Based on the data, while the IC_50_ value at 24 h post-incubation was lowest for the free drug, rDOX had the lowest IC_50_ values at 72 h, suggesting that by allowing sufficient release, the nanoparticle formulations can enhance the therapeutic effect induced by the parent drug. Furthermore, based on the poor release data and IC_50_ values observed for cDOX, it was excluded from further studies.

**Figure 5:**
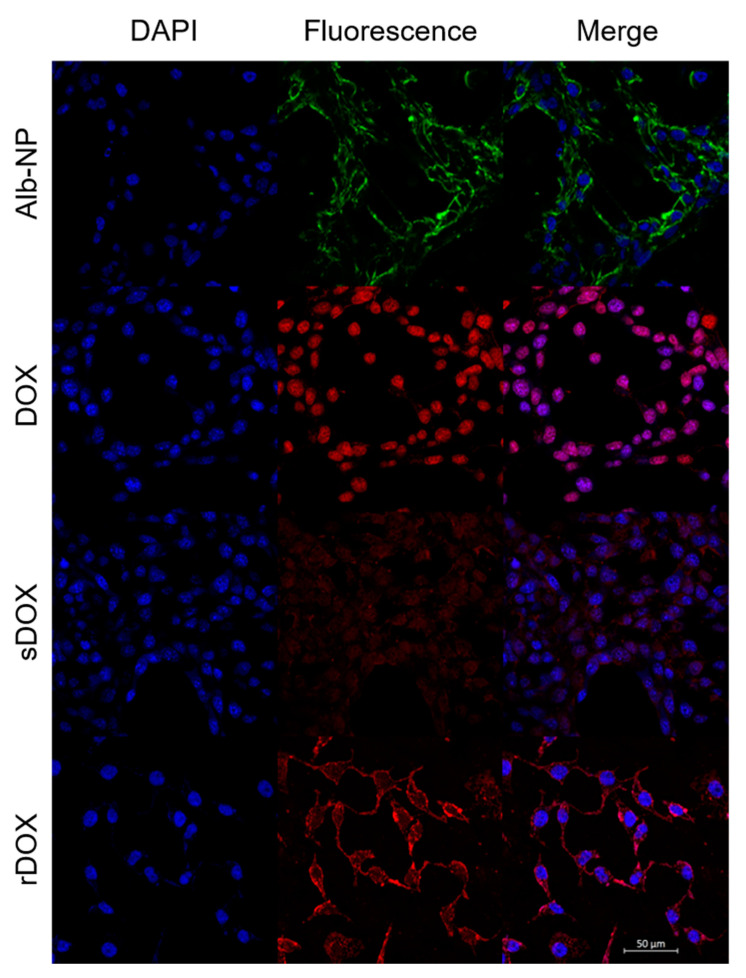
Confocal images of MDA-MB-231 cells treated with different albumin nanoparticles. Images were taken two hours after initial incubation to maximize the internalization of the nanoparticles while minimizing the cytotoxic effects of DOX on cell morphology. Scale bar: 50 μm.

Next, we had prepared slides for examination with confocal microscopy. MDA-MB-231 cells were incubated with Cy5.5-labeled Alb-NPs, DOX, sDOX, and rDOX for up to 24 h. Because the presence of DOX distorted cell morphology and viability, we decided to evaluate the endocytosis of the drug and the nanoparticles at 2 h after incubation, before DOX initiated the necrotic effects. Based on the confocal pictures taken, the bare albumin nanoparticles were able to localize within the tumor cells (Figure 5). Unlike that of the free drug, the distribution of DOX within the cell was not localized at the nucleus at the 2 h time point but rather distributed through the cell cytoplasm, which is consistent with the drug release data showing that 24 h was required for DOX to be fully released from the albumin nanoparticles. The exact mechanism behind the endocytosis of the albumin nanoparticles into the cells remains to be elucidated.

### 2.5. Evaluation of Ultrasound Treatment and Biodistribution

**Figure 6:**
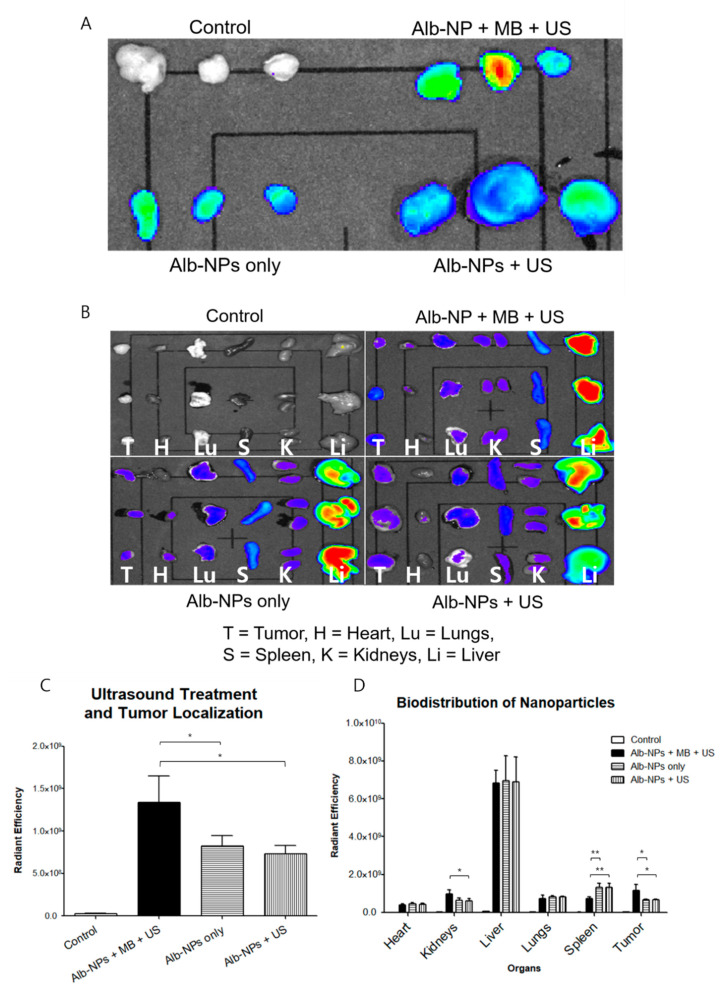
Biodistribution profiles of albumin nanoparticles in murine tumor models. (**A**) The effects of ultrasound treatment on the enhancement of albumin nanoparticle localization at the tumor were evaluated with the In Vivo Imaging System (IVIS) Spectrum 24 h after intravenous injection. (**B**) The biodistribution of albumin nanoparticles across major organs (the heart, kidneys, liver, lungs, spleen, and tumor) was evaluated with the IVIS Spectrum 24 h after intravenous injection. (**C**,**D**) represent the calculated fluorescence data from the respective organs. Values are mean ± SD (*n* = 3), * *p* ≤ 0.05, ** *p* ≤ 0.01.

To establish the localization of nanoparticles at the tumor site and the effects of ultrasound on enhanced drug delivery, we prepared Cy5.5-labeled Alb-NPs and rAlb-NPs to be administered intravenously into the subcutaneous xenograft mouse models. We first attempted to establish the ultrasound treatment conditions by evaluating the amount of Cy5.5-labeled albumin nanoparticles accumulated at the treated tumors. Four experimental groups, including the negative control group receiving saline, mice receiving Cy5.5-Alb-NPs, mice receiving Cy5.5-Alb-NPs with ultrasound exposure, and those receiving Cy5.5-Alb-NPs with microbubbles and ultrasound treatment were prepared (*n* = 3 per experimental group). At 24 h after injection, the mice were sacrificed and the tumors were isolated for In Vivo Imaging System (IVIS) Spectrum analysis. The group of mice that received the nanoparticles with complete ultrasound treatment (ultrasound + microbubbles) had significantly higher fluorescence from the tumors compared to those that received only the nanoparticles or nanoparticles with ultrasound only (Figure 6A). Intrigued by this result, we then assessed the effects of ultrasound treatment on the general biodistribution. The same experimental groups were prepared using new sets of mice (*n* = 3 per experimental group), and various organs including the heart, kidneys, lungs, liver, and spleen and the implanted tumor were collected. Organ-based analysis revealed that the injected nanoparticles had localized mostly in the liver, while some fluorescence was observed in the spleens, kidneys, and the tumors as well (Figure 6B). Similar to in the previous experiments, enhanced accumulation of fluorescence signals was observed in the tumors of the mice that received the complete ultrasound treatment.

### 2.6. In Vivo Efficacy and Safety Study

**Figure 7:**
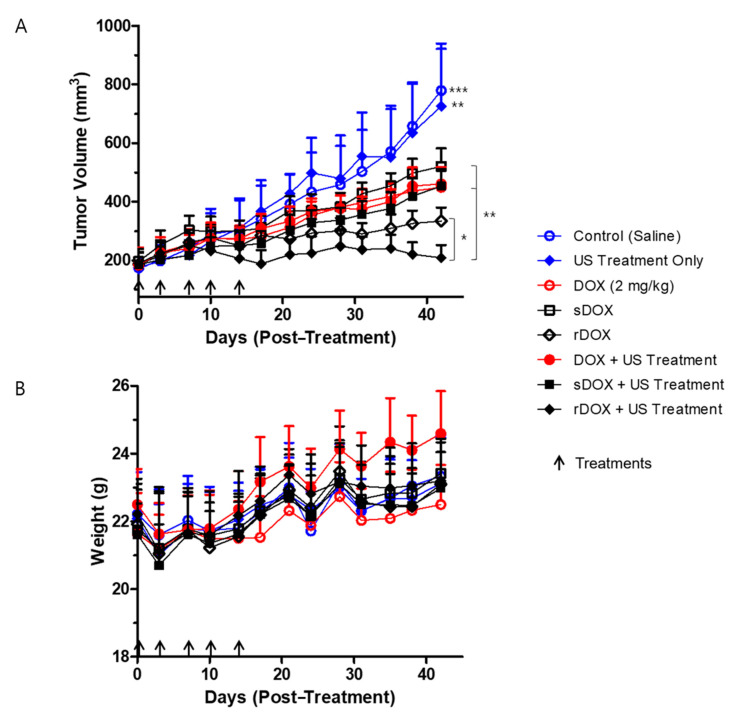
Efficacy of treatment protocols against tumor growth, and the changes in body weight. (**A**) Significant differences between the control groups (groups that received saline and ultrasound treatment only) and the treatment groups (DOX/DOX + Ultrasound (US), sDOX/sDOX+US, rDOX/rDOX + US), DOX/sDOX and rDOX groups, and rDOX and rDOX+US were observed. A concentration equivalent of 2 mg/kg DOX was used per treatment. (**B**) No significant changes in the body weights of the different groups were observed during the entire experiment. Values are mean ± SD (*n* = 5). * *p* ≤ 0.05, ** *p* ≤ 0.01, *** *p* ≤ 0.001.

Lastly, we prepared a set of experiments to evaluate the therapeutic index of the DOX-loaded albumin nanoparticles. Eight experimental groups bearing MDA-MB-231 breast cancer models were prepared as described in the Materials and Methods section: (i) negative control injected with saline, (ii) ultrasound treatment only, (iii) DOX (2 mg/kg), (iv) sDOX only (2 mg/kg), (v) rDOX only (2 mg/kg), (vi) DOX + US, (vii) Sdox + US, and (viii) rDOX + US. A dose of 2 mg/kg of DOX was used for all therapeutic protocols unless described otherwise. First, the ultrasound treatment (focused ultrasound + microbubble) itself did not have a significant effect on the tumor growth, as the tumor growth in both the control group and the ultrasound only group was unchanged (Figure 7A). Furthermore, while all formulations that contained DOX had significantly retarded the tumor growth, rDOX was the most effective. Specifically, when rDOX was complemented with focused ultrasound, the therapeutic efficacy was maximized. It is also worth noting that all the protocols, including those that included DOX, did not induce significant changes in the weights of the mice (Figure 7B).

**Figure 8:**
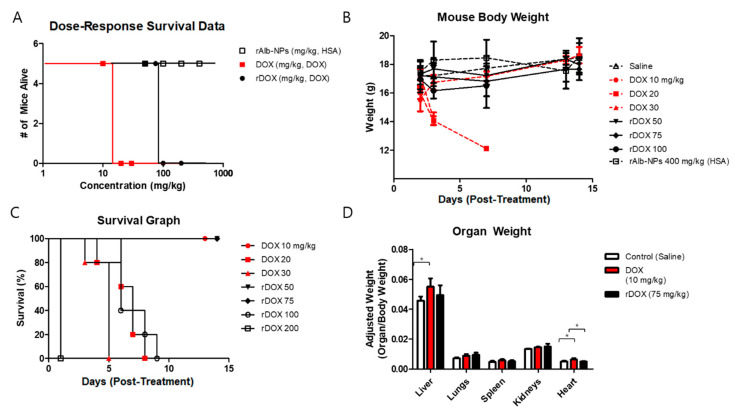
Assessment of safety profiles of albumin nanoparticles and DOX-loaded albumin nanoparticles. (**A**) Dose–response survival was obtained by using different concentrations of the rAlb-NPs, DOX, and rDOX. The LD_50_ for DOX was approximately 15 mg/kg, and that for rDOX was 87.5 mg/kg. The LD_50_ for rAlb-NPs could not be defined because no mice had died up to 400 mg/kg (human serum albumin (HSA) concentration). (**B**) The mouse group that received less than the LD_50_ values consistently gained weight, while those receiving significant doses had a dramatic reduction in body weights. (**C**) The mouse group that received significant doses of the treatment protocol had died within a week of acute injection. (**D**) The analysis of organ weights showed that there was a significant difference in the heart, an organ known to be affected by DOX. Additionally, the livers of those that received 10 mg/kg DOX weighed more than those of the control mice. Values are mean ± SD (*n* = 5). * *p* ≤ 0.05.

In addition to monitoring the mice’s weights during the efficacy studies, we also tried to assess the safety by performing different experiments to evaluate single-dose acute toxicity, dose–response survival, body weight, and organ weights. Based on the in vitro cytotoxicity and the in vivo efficacy data, rAlb-NPs, free DOX, and rDOX formulations were compared. First, when different concentrations of the three formulations were examined, the LD_50_ of the rAlb-NPs was over 400 mg/kg (HSA concentration), as none of the mice had died or showed a significant clinical symptom. On the other hand, the LD_50_ for DOX and rDOX were 15 and 87.5 mg/kg (DOX concentration), respectively, suggesting that the nanoformulation had significantly improved the toxicity profiles compared to that of the free drug (Figure 8A). Similarly, the groups that were intravenously administered a single dose of more than 20 mg/kg of free DOX, or 100 mg/kg of rDOX (DOX concentration) had died within a week of receiving the treatment protocols (Figure 8B), while those that received less than that amount had survived for more than two weeks without significant changes in their body weights (Figure 8C). Lastly, the approximated maximum tolerated doses for DOX (10 mg/kg) and rDOX (75 mg/kg) administered and the major organs from these experimental mice (the liver, lungs, spleen, kidneys, and heart) were collected for analysis. The weights of the livers and hearts of the mice that received 10 mg/kg DOX were significantly larger than those of the control mice, while the weights of the hearts in the mouse cohort that received rDOX were significantly lower than those that received DOX injection as well (Figure 8D).

## 3. Discussion

Many chemotherapies that are used in clinics today are flawed due to the potential toxicity associated with them. Anthracyclines such as doxorubicin that intercalate between the DNA base pairs and prevent DNA replication are extremely efficient in exerting cytotoxic effects against cancerous cells, but they are indiscriminative and damage normal cells as well [20,21]. The known side-effects of doxorubicin include dose-dependent cardiac toxicity from dilated cardiomyopathy, which can lead to congestive heart failures [21,22,23]. Accordingly, various efforts have been combined to address two central aims in drug-mediated therapies: maximizing drug specificity by enhancing the amount of drug exposure only at the target of interest, and minimizing drug toxicity by reducing the amounts of drugs that reach non-targeted regions (i.e., normal cells) and elicit non-specific damage. Nanoparticle formulations based on organic materials such as phospholipids, polymers, or proteins have been used in the past, but many of these formulations were based on weak interactions between the constituents, leaving their stability to be questioned [24,25]. Those that use covalent crosslinking to enhance the stability of the nanoparticles have been also researched, but the extent to which crosslinkers could have potential non-specific interactions have not been considered. The amine groups present on DOX have been often utilized as a target for stable crosslinking onto nanoformulations [26,27,28]. However, while this covalent bonding would be useful for stability, the efficacy and toxicity profiles of the drug would then need to be re-evaluated completely because the molecular characteristics would change dramatically.

To address these challenges, we present a novel combination of albumin-based nanoparticle formulations with focused ultrasound treatment to greatly improve both efficacy and toxicity profiles. Such nanoformulations are first reacted with sodium borohydride to reduce active aldehyde groups on the surface to alcohols, minimizing their potential toxicity and reactivity. As the active aldehyde groups are eliminated, the amine group on DOX will not react covalently with the particles’ surface, ensuring that the loaded DOX is bound on the reduced nanoparticles mainly by the reversible electrostatic forces, unlike for the regular albumin nanoparticles. Such a reduction step could therefore allow the improved controlled release of the therapeutic payload upon specific triggers such as acidic pH without altering the structure of the contents themselves involved in the breakdown of covalent bonds. The release of DOX from the albumin nanoparticles, especially from the reduced ones, was therefore maximized when the pH of the environment was acidic—as found in tumor microenvironments, where the pH is reported to be <6.5 [29]—but not in regions with physiological conditions, as described in Figure 3. The data from confocal microscopy also support this hypothesis, as it was observed that stronger fluorescence signals were observed with rDOX at 2 h post-incubation than with sDOX, which we speculate to be induced by the improved release of the drug. Cardiotoxicity, including changes in myocardial structure and function to severe cardiomyopathy, is a major side-effect associated with the administration of DOX at high doses. One of the clinical symptoms associated with such heart conditions is cardiac enlargement, which can cause heart failure. Based on our organ-based toxicity data, cardiac enlargement was observed in the mice with one-time administration of 10 mg/kg of free DOX, but not in the mice that received 75 mg/kg DOX loaded on albumin nanoparticle formulations. In addition, the rAlb-NP vehicle itself did not cause any significant changes in the survival, overall body mass, individual organs, or observable behavioral patterns at up to 400 mg/kg, suggesting that the albumin nanoparticles themselves are not toxic.

We also employed focused ultrasound to enhance the accumulation of nanoparticles where the local release of DOX would be facilitated. The use of ultrasound to improve local drug delivery is a relatively well-established technique that is currently being applied in numerous preclinical and clinical models, including brain, breast, and pancreatic cancers. By temporarily disrupting the endothelial linings by microbubble-assisted cavitation, focused ultrasound can further augment the enhanced permeability and retention effects and increase the number of nanoparticles available locally, as observed from the IVIS Spectrum-based fluorescence data. Combining both the enhanced localization and improved control of DOX release, we observed a significant improvement in the anti-cancer activities when rDOX was used in combination with focused ultrasound, compared to standalone DOX therapies or rDOX therapies. However, unlike rDOX vs. Rdox + US, we did not observe a statistically significant increase in anti-cancer effects for DOX vs. DOX+US or sDOX vs. sDOX + US. We hypothesized two potential explanations for the observed phenomena based on experimental evidence: (1) Ultrasound-mediated drug delivery is most effective against tumors with low permeability [30,31]. Because DOX molecules themselves were effective (i.e., able to reach the tumor to elicit anti-cancer effects) in the mouse models used in this study, introducing ultrasound treatment to enhance permeability was not as effective as in other models such as pancreatic cancer models [15] reported in the literature. (2) Nanoparticles are much bigger than the drug molecules themselves; therefore, using ultrasound to introduce extra fenestrations in the vasculature for extravasation into the tumor interstitial space would be much more beneficial for the nanoparticles than the drugs, as observed in rDOX vs. rDOX + US. Nevertheless, because sDOX has poor release profiles when compared to rDOX, not all sDOX molecules that reach the tumor microenvironment may fully release their payload, reducing their therapeutic efficacy. Nevertheless, the tremendous increase in the therapeutic efficacy and toxicity profiles presented by rDOX and its synergistic effects with focused ultrasound shows promise for the next generation of drug delivery platforms using fully biocompatible albumin-based nanoparticles in combination with an external stimulus. Further studies on optimizing the therapeutic conditions, including the drug dose, schedule, ultrasound parameters, drug combinations, and potential resistance, are required to maximize such potential and introduction into clinical settings.

## 4. Materials and Methods

### 4.1. Reagents and Equipment

Human serum albumin (HSA) was acquired from SK Chemicals (SK Chemicals, Seongnam, Korea). Doxorubicin (DOX) was acquired from Boryung Pharmaceutical (Boryung Pharmaceutical, Seoul, Korea). Sodium borohydride (NaBH_4_), sodium cyanoborohydride (NaCNBH_3_), silver nitrate solution, and glutaraldehyde (GTA) were purchased from Sigma Aldrich (St. Louis, MO, USA). SonoVue^®^ microbubbles (MB) were acquired from Bracco (Bracco, Italy). Zolazepam (Zoletil^®^) was obtained from Virbac (Virbac, Carros, France), and xylazine hydrochloride (Rompun 2%) was acquired from Bayer (Bayer Korea, Seoul, Korea). The 1260 Infinity II LC system was acquired from Agilent Technologies (Agilent Technologies, Santa Clara, CA, USA). The VIFU 2000^®^ was acquired from Alpinion Medical Systems (Alpinion Medical Systems Co., Ltd., Seoul, Korea).

### 4.2. Preparation of Albumin Nanoparticles

Human serum albumin nanoparticles (Alb-NPs) were synthesized based on a modified desolvation protocol [18]. One gram of HSA was added to 340 mL of distilled water, and 2000 μL of 1 M sodium hydroxide (NaOH) was added dropwise to adjust the pH. To induce the agglomeration of HSA, ethanol was added dropwise under stirring conditions (500 rpm) at room temperature until turbidity of the solution was obtained. Excess 8% GTA solution (1000 μL) was added to the HSA aggregates and reacted for 24 h under stirring conditions to ensure complete crosslinking. After crosslinking was completed, the solution was centrifugated at 15,000 rpm for 15 min at 4 °C, and the pellet was re-dispersed with distilled water three times to remove unbound chemicals and then kept in a refrigerator until further use.

### 4.3. Preparation of Reduced Alb-NPs (rAlb-NPs)

One hundred micrograms of Alb-NPs was added to 20 mL of ethanol, and 100 μL of reducing agent (sodium borohydride and sodium cyanoborohydride, respectively) was added under stirring (500 rpm) overnight. The pH was adjusted to 7.5~8.5 with 1 M sodium hydroxide. The solution was centrifugated at 15,000 rpm for 15 min at 4 °C, and the pellet was re-dispersed with distilled water three times to remove unbound chemicals and then kept in a refrigerator until further use. In addition, the rAlb-NPs were checked for purity using the ICP-AES methodology.

### 4.4. Characterization of the Alb-NPs and rAlb-NPs

The hydrodynamic size, polydispersity, and zeta potential of the prepared nanoparticles were measured using the dynamic light scattering (DLS) method (Zetasizer Nano ZS90; Malvern Instruments, Malvern, UK). The detection of free aldehyde in both groups was performed using Tollens’ reagent according to the manufacturer’s protocol. In short, 0.3 M NaOH solution was added dropwise to 0.3 M silver nitrate solution until a silver precipitate formed, to which 3 M ammonia solution was added dropwise until the solution’s color became transparent. Ten milliliters of the prepared Tollens’ reagent was added to 10 mL of 1 mg/mL of Alb-NPs or rAlb-NPs (reduced by NaBH_4_ or NaCNBH_3_) and reacted for 6 or 24 h, and the change in color was observed. The detection of boron was evaluated using ICP-AES at the National Center for Inter-University Facilities (Seoul National University, Korea). The morphology and size of the nanoparticles were further studied with transmission electron microscopy (TEM) and scanning electron microscopy (SEM) for further analysis at the National Center for Inter-University Facilities, Seoul National University (South Korea). Last, the stability of the nanoparticles was analyzed by characterizing the stored nanoparticles every month.

### 4.5. Preparation of DOX-Loaded Albumin Nanoparticles

We have evaluated three different methods of loading DOX onto the albumin nanoparticles. The first method involved encapsulating DOX before ethanol addition during the desolvation process. DOX was added in weight ratios of 1:3, 1:5, and 1:10 of DOX to HSA. The HSA–DOX mixture was stirred for an hour; then, ethanol was added dropwise until the mixture turned turbid. GTA (8%) was added, and the reaction was performed for 24 h under stirring conditions (500 rpm). After the crosslinking of the HSA and DOX mixture was completed, the solution was centrifuged at 15,000 rpm for 15 min at 4 °C and the pellet was re-dispersed with distilled water three times to remove unbound chemicals. The supernatants from each wash were collected and analyzed with HPLC to calculate the loading efficiency for DOX. The albumin nanoparticles encapsulating DOX were termed cDOX.

The second and the third methods of loading DOX involved first synthesizing albumin nanoparticles based on the desolvation and reduction methods outlined previously and then coating the Alb-NPs with DOX. A 90 mg amount of the albumin nanoparticles in 5 mL of solution (prepared according to Section 4.2 and Section 4.3) was reacted with DOX at different weight ratios (*w*/*w*% of 1:1, 1:2, 1:3, 1:5, and 1:10 DOX/albumin nanoparticles) for 24 h at room temperature in the dark under stirring conditions (500 rpm). The DOX–albumin nanoparticle mixtures were topped up to 10 mL using deionized water and adjusted to pH 8.5 to prevent aggregation during the reaction. The solution was centrifugated at 18,000 rpm for 15 min at 4 °C, and the pellet was re-dispersed with distilled water three times to remove unbound chemicals. The supernatants from each wash were collected and analyzed with HPLC to calculate the loading efficiency for DOX. The albumin nanoparticles coated with DOX were termed sDOX and rDOX (reduced).

### 4.6. In Vitro Kinetics of DOX Release from Albumin Nanoparticles

cDOX, sDOX, and rDOX were added at 50 mg/mL into membrane dialysis bags (cutoff Molecular Weight (MW), 2000), which were transferred into beakers containing 50 mL of buffer solutions prepared at various pHs (pH 7.4, 6.5, and 5.5). The solutions were incubated at 37 °C while under mechanical stirring. At each time point (1, 3, 6, 9, and 24 h after addition), 1 mL aliquots of the solutions were transferred to 1.5 mL Eppendorf tubes and centrifuged at 15,000 rpm for 30 min at 4 °C to separate the released DOX from the nanoparticles. The amount of DOX released from the nanoparticles was measured by analyzing the supernatants with HPLC at 260 nm to quantify the amount of DOX released.

### 4.7. Cell Culture

Human triple-negative breast cancer cell line MDA-MB-231 and murine macrophage cell line Raw264.7 cells were acquired from the American Type Culture Collection (ATCC) and were cultured in Dulbecco’s Modified Eagle Medium (DMEM) supplemented with 10% heat-inactivated fetal bovine serum (FBS), 100 IU/mL penicillin, 100 mg/mL streptomycin, and 2 mM L-glutamine. Cultures were maintained in a humidified atmosphere with 5% CO_2_ at 37 °C and routinely tested for mycoplasma contamination. Cells were sub-cultured once they reached 80% confluency, determined by the trypan blue dye exclusion method.

### 4.8. Cell Viability Assay

The CellTiter 96^®^ AQueous One Solution Cell Proliferation Assay (MTS) was used to assess the effects of nanoparticles on cell viability. MDA-MB-231 and Raw264.7 cells were seeded on 96-well plates at a density of 5 × 10^3^ cells per well and incubated overnight. First, the effects of bare albumin nanoparticles on cell viability were evaluated by adding various concentrations of Alb-NPs and rAlb-NPs to both Raw264.7 and MDA-MB-231 cells. In addition, the cytotoxic effects of the drugs were examined by adding DOX, cDOX, sDOX, and rDOX into cells and incubating for up to 72 h. Cells were removed from the incubator at designated time points, and their viability was evaluated against the phosphate-buffered saline (PBS) controls using the MTS solution to derive approximate IC_50_ values.

### 4.9. Confocal Laser Scanning Microscopy

MDA-MB-231 cells were seeded on 8-well chamber slides (Nunc™ Lab-Tek™ II Chamber Slide™ System, Thermo Fisher Scientific, Waltham, MA, USA) at a density of 3 × 10^4^ cells per well and incubated overnight. Albumin nanoparticles encapsulating Cy5.5-NHS ester dye (Lumiprobe, Hallandale Beach, FL, USA) were prepared by adding the fluorophore instead of DOX during the desolvation process described in Section 4.2 (Cy5.5-Alb-NPs). On the next day, the cells were treated with various concentrations of Cy5.5-labeled and DOX-loaded albumin nanoparticles and were further incubated for a varying period. Once incubation was completed, the cells were fixed for 15 min with 4% formaldehyde and counter-stained with 4′,6-diamidino-2-phenylindole dyes (DAPI, Thermo Fisher Scientific, Waltham, MA, USA). During the fixation and staining processes, the cells were washed with fresh PBS. The images were acquired using a confocal microscope (Carl Zeiss, Inc., Oberkochen, Germany), using the excitation/emission wavelengths of 358/461, 470/595, and 684/710 nm for DAPI, DOX, and Cy5.5, respectively.

### 4.10. In Vivo Study

Immunocompetent female BALB/c mice and immunodeficient BALB/c nude mice that were 6–8 weeks old were purchased from Orient Bio (Seoul, Korea) for the toxicity and efficacy studies, respectively. The mice were acclimated for a week before the start of the study and were maintained at standard conditions in specific pathogen-free (SPF) environments: 25 ± 2 °C temperature, 50 ± 10% relative humidity, and 12 h light/12 h dark. All mice were fed with sterilized standard mouse chow and water ad libitum. After the acclimatization periods, 1 × 10^6^ MDA-MB-231 cells suspended in Matrigel (Corning, Tewksbury, MA, USA) were injected into the right flank regions of the nude mice. Once the tumor volume had reached ~150 mm^3^, the mice were randomly sorted for the treatment. The tumor sizes were monitored with a digital caliper, and the volumes were calculated according to the formula width^2^ × length × 0.5. All the in vivo protocols (Approval Number: BA1906-275/046-01) were verified according to the guidelines of the Seoul National University Bundang Hospital.

### 4.11. Ultrasound Treatment Protocols

A focused ultrasound system (VIFU 2000^®^, Alpinion Medical Systems, Seoul, Korea) was used for focused ultrasound (FUS) treatments: a 1.1 MHz single-element, spherically focused transducer with a central circular opening of 40 mm in diameter, creating a focal zone of 1.3 × 1.3 × 9.2 mm with a center frequency of 1.1 MHz at −6 dB, was controlled with a 3D target position system and ultrasound guidance to precisely deliver therapeutic ultrasound to the target. A degassing chamber was used to ensure that gas levels in the system were kept to a minimum (≤4 ppm) during the treatment. After injecting the mice with therapeutic formulations (drug + microbubbles), pulsed FUS beams with the acoustic parameters of a 1.1 MHz frequency, 20 watts of power, a 40 Hz pulse repetition frequency, a 5% duty cycle, 5 s of ultrasound exposure per spot, and a 2 mm spot distance were applied at the tumor.

### 4.12. Biodistribution and IVIS Spectrum

Albumin nanoparticles encapsulating Cy5.5 dyes (Cy5.5-Alb-NPs) were prepared according to the protocol described in Section 4.9. Four experimental groups—(i) negative control injected with saline, (ii) Cy5.5-Alb-NPs, (iii) Cy5.5-rAlb-NPs, and (iv) Cy5.5-Alb-NPs + MB + FUS—were prepared. For the treatments, 200 μL amounts were injected intravenously into the MDA-MB-231 tumor-bearing mice. After 24 h, the mice were sacrificed and the Cy5.5 fluorescence signals from the tumors were analyzed using the In Vivo Imaging System (PerkinElmer, Waltham, MA, USA).

### 4.13. Experimental Groups and Protocols for Efficacy Study

The experimental groups for the efficacy study were defined as follows: (i) negative control injected with saline, (ii) DOX (2 mg/kg), (iii) sDOX only (2 mg/kg), (iv) rDOX only (2 mg/kg), (v) MB + FUS, (vi) sDOX + MB + FUS, and (vii) rDOX + MB + FUS. Before the treatment, intraperitoneal general anesthesia was administered using a mixture of 30 mg/kg Zoletil and 10 mg/kg Rompun 2%. All experimental groups received their treatments intravenously, and those that concurrently received ultrasound treatment were additionally administered with 200 μL of SonoVue (1 × 10^8^ MB/mL) immediately after the injection of the respective treatments. Each group received five treatments on Days 3, 7, 10, 14, and 17, and the tumor sizes were monitored biweekly for up to 4 weeks after the final treatment.

### 4.14. Experimental Groups and Protocols for Toxicity Study

The experimental groups for the acute toxicity study were defined as follows: (i) negative control injected with saline, (ii) DOX (10 mg/kg), (iii) DOX (20 mg/kg), (iii) DOX (30 mg/kg), (iv) rDOX (50 mg/kg DOX), (v) rDOX (75 mg/kg), (vi) rDOX (100 mg/kg), and (vii) rDOX (200 mg/kg). Each group received a single intravenous injection of 200 μL of the respective treatment. The mice were monitored for two weeks following the injection, and their weights and conditions were recorded at Days 2, 3, 7, 13, and 14 post-injection. At Day 14, the surviving mice were sacrificed, and their organs—liver, lungs, spleen, kidneys, and heart—were collected for further analysis.

### 4.15. Statistical Analysis

Data are expressed as mean ± standard deviation (SD). Nonlinear regression analysis was conducted to calculate the IC_50_ values, and one-way ANOVA with Tukey’s post hoc analysis was used to compare experimental groups (GraphPad Prism 5.0, San Diego, CA, USA). Probability (*p*) values of <0.05 were considered as statistically significant.

## 5. Conclusions

In this work, human serum albumin was crosslinked using GTA to form uniform, spherical nanoparticles with unique abilities to load DOX, a widely used anticancer drug. In addition, we further modified this nanoformulation by using reducing agents to remove unreacted aldehydes from the surface, minimizing the potential toxicity associated with non-specific interactions enhancing the controlled release of the drug payload upon the external trigger. Furthermore, focused ultrasound was applied to enhance the accumulation of nanoparticles at the targeted local tumor, allowing DOX-dependent cancer cell death and superior tumor inhibitory effects compared to those achieved with the free drug or DOX-loaded, non-reduced albumin nanoparticle formulations. Additionally, we were able to observe a higher safety margin, highlighted by a much-improved maximum tolerated dose and reduced cardiac stress. Accordingly, the development of albumin-based nanoparticles holds great potential for anticancer therapy, and we believe further optimization of the platform could lead their way into clinical settings.

## Figures and Tables

**Table 1:** The average size and the zeta potential values of albumin nanoparticles (Alb-NPs, rAlb-NPs, sDOX, rDOX, and cDOX) (*n* = 3).

Nanoparticles	Mean Size (Mean ± SD, nm)	Zeta Potential (mV)
Alb-NPs	146 ± 37.5	−51.3 ± 2.1
rAlb-NPs	142 ± 31.1	−24.4 ± 2.8
cDOX	151 ± 21.5	−27.5 ± 1.1
sDOX	144 ± 28.2	−21.6 ± 1.6
rDOX	146 ± 31.8	−14.7 ± 0.8

**Table 2:** The stability of albumin nanoparticles at 4 °C over time evaluated by changes in their size and zeta potential (*n* = 3).

Nanoparticles	Days	Mean Size (Mean ± SD, nm)	Zeta Potential (mV)
Alb-NPs	1	146.8 ± 40.1	−50.1 ± 3.75
	3	142.9 ± 38.7	−51.4 ± 4.17
	7	137.3 ± 30.4	−46.7 ± 3.27
	14	151.7 ± 28.0	−49.5 ± 5.81
	30	148.1 ± 31.7	−47.9 ± 2.37
	90	145.8 ± 38.1	−50.5 ± 3.04
	180	150.7 ± 41.4	−48.1 ± 2.90
rAlb-NPs	1	142.6 ± 45.8	−27.2 ± 3.75
	3	148.6 ± 37.8	−28.6 ± 5.85
	7	148.2 ± 29.4	−26.3 ± 3.10
	14	145.9 ± 34.1	−21.8 ± 2.94
	30	141.8 ± 33.8	−31.7 ± 4.51
	90	148.1 ± 30.4	−27.2 ± 4.48
	180	146.4 ± 32.1	−28.1 ± 3.41

**Table 3:** The loading efficiency and extraction of DOX on/from albumin nanoparticles (*n* = 3).

Nanoparticles	Ratio (*w*/*w*)	Loading Efficiency (%)	Extraction @ pH 1 (%)
cDOX	10:1	67.1 ± 5.87	21.74 ± 5.12
	5:1	Aggregation	Aggregation
	3:1	Aggregation	Aggregation
sDOX	10:1	95.2 ± 5.21	50.1 ± 3.75
	5:1	93.4 ± 3.73	51.4 ± 5.17
	3:1	94.7 ± 4.71	46.7 ± 5.27
rDOX	10:1	95.1 ± 3.51	97.4 ± 4.27
	5:1	Aggregation	Aggregation
	3:1	Aggregation	Aggregation
